# The Role of Natural and Synthetic Flavonoids in the Prevention of Marine Biofouling

**DOI:** 10.3390/md22020077

**Published:** 2024-02-02

**Authors:** Daniela Pereira, Madalena Pinto, Joana R. Almeida, Marta Correia-da-Silva, Honorina Cidade

**Affiliations:** 1 Laboratory of Organic and Pharmaceutical Chemistry, Department of Chemical Sciences, Faculty of Pharmacy, University of Porto, Rua de Jorge Viterbo Ferreira 228, 4050-313 Porto, Portugal; 2 Interdisciplinary Centre of Marine and Environmental Research (CIIMAR), University of Porto, Edifício do Terminal de Cruzeiros do Porto de Leixões, Avenida General Norton de Matos, S/N, 4450-208 Matosinhos, Portugal

**Keywords:** flavonoids, natural, synthesis, biofouling, antifouling activity

## Abstract

Marine biofouling is a major concern for the maritime industry, environment, and human health. Biocides which are currently used in marine coatings to prevent this phenomenon are toxic to the marine environment, and therefore a search for antifoulants with environmentally safe properties is needed. A large number of scientific papers have been published showing natural and synthetic compounds with potential to prevent the attachment of macro- and microfouling marine organisms on submerged surfaces. Flavonoids are a class of compounds which are highly present in nature, including in marine organisms, and have been found in a wide range of biological activities. Some natural and synthetic flavonoids have been evaluated over the last few years for their potential to prevent the settlement and/or the growth of marine organisms on submerged structures, thereby preventing marine biofouling. This review compiles, for the first-time, natural flavonoids as well as their synthetic analogues with attributed antifouling activity against macrofouling and microfouling marine organisms.

## 1. Introduction

Marine biofouling is a consequence of the settlement and accumulation of adhesive marine organisms’ forms on submerged surfaces, namely on ships, causing huge concerns for the maritime industry, the marine environment, and human health. The process of marine biofouling involves different stages, starting with the adsorption of proteins, glycoproteins, or polysaccharides, constituting the conditioning film, which is followed by the deposition of marine bacteria, leading to the formation of a bacterial biofilm. Consequently, extracellular polymeric substances are secreted by the bacterial biofilms, leading to the settlement and accumulation of diatoms and macroalgal spores on the surface of the biofilm, and subsequently to the settlement of macrofouling organisms, including mussels, barnacles, and algae [1,2]. This phenomenon causes huge economic impacts for maritime industries, since the increase in a ship’s roughness leads to an increase in fuel consumption and consequently to extra fuel costs. Moreover, the damage to ships due to discoloration and corrosion are also notorious, and a longer drydock period is necessary to repair the ship [3]. Marine biofouling is also responsible for environmental and health concerns, as it promotes an increase in the emission of polluting gases and the spread of invasive species to other geographical locations, threatening the native species, reducing the biodiversity, and causing the introduction of diseases [3,4,5]. Considering the huge impact of marine biofouling, some antifouling strategies have been adopted to prevent this phenomenon. Since the 1960s, the incorporation of tributyltin (TBT) in marine coatings were used by the maritime industries worldwide, as TBT was able to control a broad spectrum of fouling organisms. However, TBT has been demonstrated to cause impairments in the growth, development, reproduction, and survival of many marine species, and therefore, the use of this biocide was completely banned by the International Maritime Organization in 2008 [6]. As an alternative to the use of TBT and other organotins, some old-fashioned techniques, namely using copper and zinc, and booster biocides, including Irgarol 1051, Zinc pyrithione, Chlorothalonil, and Sea Nine 211, have been used in marine coatings. However, although some of these molecules were described as environmentally friendly biocides, several toxic effects have been attributed to these alternative biocides [7].

**Figure 1:**
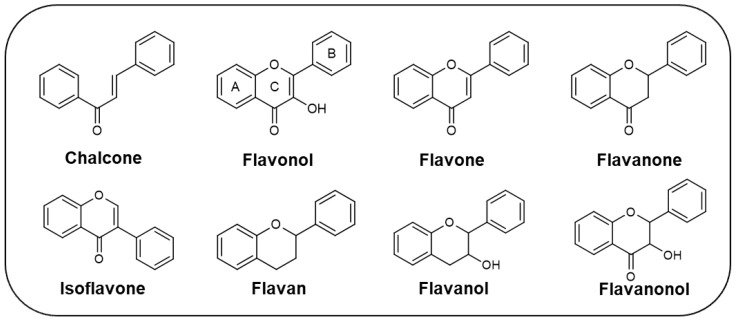
Examples of subclasses of flavonoids.

Since the antifouling compounds used have proven to be harmful to the marine environment, the search for new more sustainable alternatives is urgent. One of these strategies can be the use of natural and nature-inspired compounds, namely flavonoids, which have shown interesting antifouling activity when tested against some biofouling marine organisms [8]. Flavonoids are natural polyphenols divided into several subclasses, including chalcones, flavonols, flavones, flavanones, isoflavones, flavans, flavanols, and flavanonols (Figure 1), which are not only commonly found in terrestrial plants, but also found in marine sources [9]. These compounds have been widely described over the last decades for having a wide range of biological properties, namely anticancer, antimicrobial, anti-inflammatory, antidiabetic, antioxidant, cardioprotective, and neuroprotective [9,10,11,12]. Despite the wide range of biological activities attributed to flavonoids over the last few decades, few studies reported their potential as antifoulants. To the best of our knowledge, the first paper reporting an antifouling assay using a flavonoid was carried out in 1989 [13]. Until now, 21 research papers described the antifouling potential of flavonoids obtained from marine and terrestrial sources, as well as synthetic ones, through the inhibition of the settlement of macrofouling species, namely mussels, barnacles, and algae, as well as their activity against microfouling organisms, including marine bacteria and diatoms. This article reviews, for the first time, the flavonoids with antifouling activity reported until now. The presentation of all flavonoids is organized by chemical class, and, within each chemical class, in chronological order of the year of the first report of their antifouling activity. The effect of flavonoids against the settlement/growth of some marine species involved in marine biofouling is presented, as well as the main structure–activity relationship (SAR) which is highlighted to support the design of new and optimized antifouling flavonoids.

## 2. Antifouling Flavonoids

**Figure 2:**
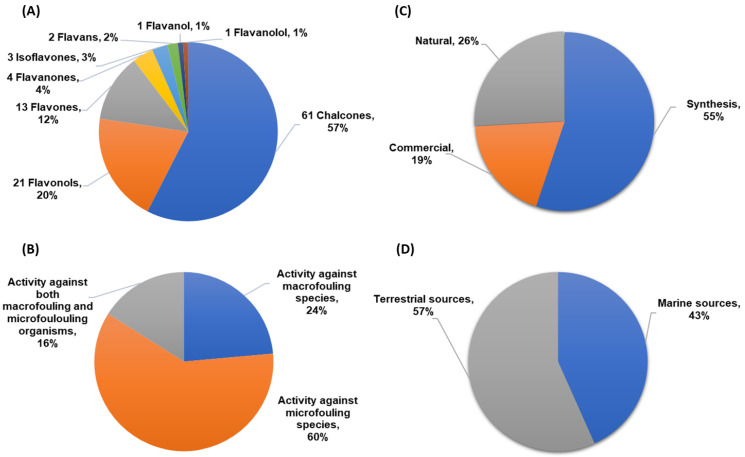
(**A**) Classes of flavonoids with antifouling activity reported in this review. (**B**) Bioactivity evaluated for antifouling flavonoids against macrofouler, microfouler, or both types of fouling organisms. (**C**) Basis of flavonoids studied for antifouling activity. (**D**) Source of natural flavonoids.

A total of 106 flavonoids with antifouling activity against macrofouling (mussels, barnacles, algae) and microfouling (diatoms, bacteria, protozoans) species have been reported, including mainly chalcones, flavonols, and flavones. Other classes of flavonoids with antifouling potential were also reported but less represented, namely flavanone, isoflavone, flavan, flavanol, and flavanonol derivatives (Figure 2A). Most of the studies (60%) were performed using microfouling species (namely marine bacteria and diatoms), and only 24% of the compounds were evaluated against macrofouling organisms, mainly mussel and barnacle species. Nonetheless, 16% of the tested compounds were evaluated against both macrofouling and microfouling organisms (Figure 2B). Considering the origin of the flavonoids reported in this review, as shown in Figure 2C, most of the compounds were obtained through synthesis in a laboratory (55%), followed by natural flavonoids (26%) and commercial compounds (19%). Among natural flavonoids, the percentage of antifouling flavonoids obtained from marine sources is almost as high as those from terrestrial sources, as shown in Figure 2D. The structure, origin, and antifouling effects of flavonoids are presented in Table S1 (Supplementary Material), which is organized in chronological order of the year of the first report of the antifouling activity.

### 2.1. Chalcones

**Figure 3:**
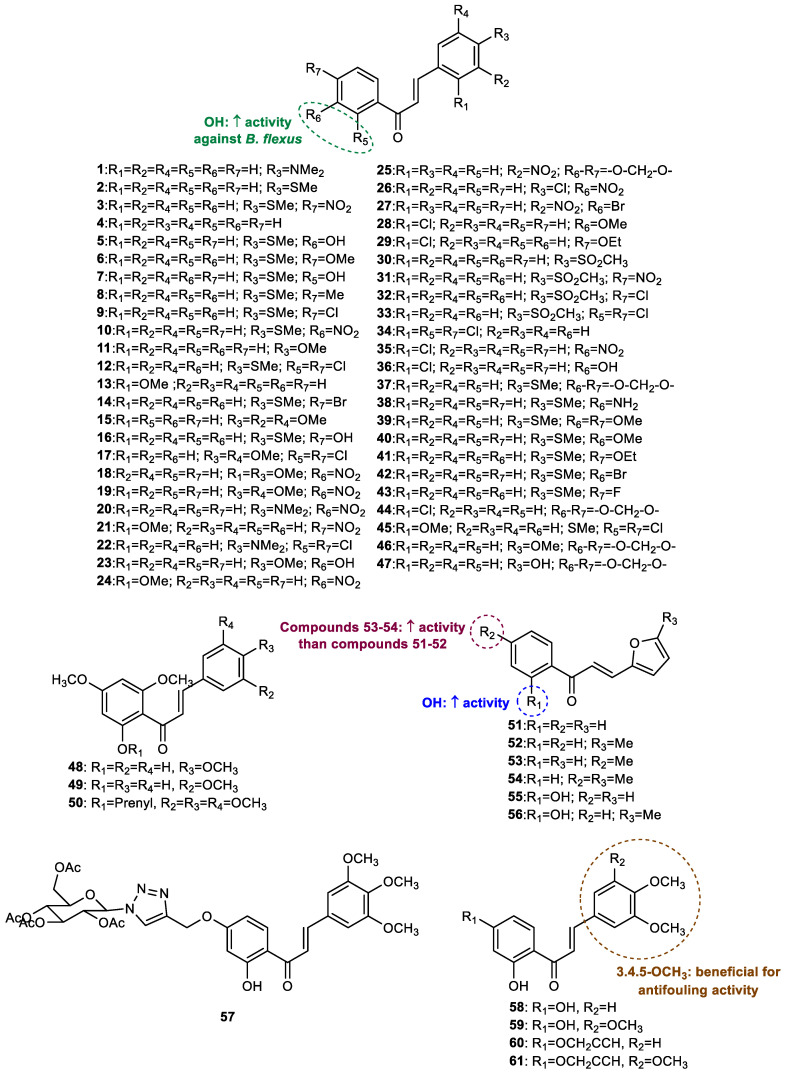
Structures of chalcones **1**–**61** with antifouling activity and SAR considerations.

A total of 61 chalcones with antifouling potential have been reported. Although this class of flavonoids is common in nature, all chalcones reported to have antifouling activity were obtained by chemical synthesis. The structures of chalcones with antifouling potential are shown in Figure 3.

Sivakumar and colleagues evaluated the antifouling activity of 47 synthetic chalcone derivatives (**1**–**47**) against three marine bacteria (*Bacillus flexus*, *Pseudomonas fluorescens,* and *Vibrio natriegens*) obtained from biofilms formed on polymer and metal surfaces immersed in ocean water [14]. All compounds were shown to be highly active against *B. flexus* bacterial growth, with minimum inhibitory concentration (MIC) values between 0.002 and 0.466 µM, *P. fluorescens* (MIC values between 0.004 and 0.133 µM), and *V. natriegens* (MIC values between 0.024 and 0.249 µM) [14]. Among tested compounds, chalcones **5**, **7**, **16**, **23**, and **36** were the most active against *B. flexus*, with MIC values of 0.002–0.031 µM. All these chalcones presented a hydroxylated ring on their A ring, which seems to be an important substituent for their antibacterial activity against *B. flexus*. For *V. natriegens*, chalcones **31** and **32** presented the highest activity, with MIC values of 0.024 µM. All compounds showed potent activity regarding *P. fluorescens*. 

The same research group showed that 2-methoxy-2′,4′-dichlorochalcone (**45**), mixed with a marine paint and coated on polycarbonate (PC), polymethylmethacrylate (PMMA), and a glass-fiber-reinforced plastic (GFRP) surface, inhibited the marine bacterium *V. natriegens* biofilm [15]. This coated surface was also compared with surfaces coated with marine paint without any additive and the one mixed with copper (1%), and it was found that surfaces coated with the paint containing the 0.423 µM of 2-methoxy-2′,4′-dichlorochalcone were the most effective amongst these three in reducing the amount of biofilm as well as its thickness. The biofilm formation can be attributed to the presence of fimbria structures in the *V. natriegens* bacteria, which are used for attachment on surfaces, and these structures are highly enriched with proteins, which is one possible reason for its hydrophobicity. Since the coating with dichlorochalcone **45** increases the hydrophilicity of the polymeric surfaces, it decreases the formation of biofilm, which can explain the activity of this coating. Another assay showed that only about 15% of chalcone **45** was lost from the surface after 28 days, maintaining its activity in a sustained manner [15].

A series of synthetic chalcone derivatives were evaluated by us to determine their antifouling potential against marine macro- and microfouling organisms, including mussel *M. galloprovincialis* larvae, marine bacteria *Vibrio harveyi*, *Cobetia marina*, *Halomonas aquamarina*, *Pseudoalteromonas atlantica,* and *Roseobacter litoralis*, and marine diatoms *Cylindrotheca* sp., *Halamphora* sp., *Nitzschia* sp., and *Navicula* sp. [16]. Among the 16 compounds tested, three methoxylated chalcones, **48**, **49**, and **50**, showed effective anti-settlement activity against the *Mytilus galloprovincialis* mussel larvae, with EC_50_ values ranging from 7.24 to 34.63 µM. Chalcones **49** and **50** also showed growth-inhibitory activity against the bacterial strains *H. aquamarina* (with EC_50_ values of 18.67 and 18.78, respectively) and *R. litoralis* (with EC_50_ values of 4.09 and 12.34 µM, respectively). Moreover, chalcone **50**, with a prenyl group, also displayed inhibitory activity against diatoms *Cylindrotheca* sp., *Halamphora* sp., *Nitzschia* sp., and *Navicula* sp., with EC_50_ values ranging from 6.75 to 20.31 µM. An ecotoxicity assay performed against non-target organism *Artemia salina* showed that these compounds were not toxic at 50 µM [16].

Sathicq et al. reported the synthesis and antifouling evaluation of a series of seven chalcones, including the non-substituted chalcone (**4**) and six furylchalcones (**51**–**56**) [17]. These compounds were incorporated into a rosin-based antifouling coating, and acrylic panels were painted with these paints and further immersed in the sea for 45 days, and then the results were compared with a control paint (without the incorporation of chalcones). Concerning the evaluation of their antifouling activity against macrofouling species, paints containing non-substituted chalcone **4** did not show significant differences compared with the control paint, with only a reduction of approximately 20% in the presence of macrofouling organisms compared to the control paint, whereas furylchalcones **51**–**56** displayed a strong anti-macrofouling effect, showing a high reduction in the settlement of algae (filamentous red alga *Polysiphonia* sp., green alga *Ulva* sp., filamentous brown alga *Ectocarpus* sp.) as well as calcareous tubeworms (*Hydroides* sp. and *Spirorbis* sp.). In fact, paints with furylchalcones **53** and **55** showed an inhibition of almost 100% of the settlement of macrofouling species, and compounds **51**, **52**, **54**, and **56** showed only about 10%–25% of settlement of these organisms compared to the control paint. Regarding their anti-microfouling effect, all paints containing chalcones **4** and **51**–**56** were able to inhibit the settlement and growth of diatoms, namely *Achnanthes* sp., *Coscinodiscus* sp., *Grammatophora* sp., *Licmophora* sp., *Navicula* sp., *Nitzschia longissima*, *Pinnularia* sp., *Pleurosigma* sp., *Synedra* sp., and *Skeletonema costatum*, and protozoans such as *Vorticella* sp. and *Zoothamnium* sp. Compared to the control paint, which was 100% covered with diatoms and protozoans, for paints with unsubstituted chalcone and furylchalcones only from about 20% to 40% of the painted plate was covered with these fouling organisms, with furylchalcone **51** being the most active with less than 20% of the plate being covered with microfouling organisms. SAR studies showed that, in general, compounds **55** and **56**, with the presence of a 2′-hydroxy group on their A ring, showed higher activity than compounds **51**–**54** in the inhibition of the settlement of macrofouling species. Compounds **53**–**54**, with a methyl group on their A ring, also showed higher activity than compounds **51**–**52** against the settlement of macrofouling species, suggesting that the presence of methyl and hydroxy groups is beneficial to the antimacrofouling activity of this series of compounds [17].

More recently, our group reported a synthesis and antifouling activity evaluation for a series of eight triazole-linked flavonoid glycosides, as well as intermediate compounds. Among them, chalcone **57**, as well as structure-related non-glycosylated chalcones **58** and **59** and the 4′-propargylated chalcones obtained as synthetic intermediates (**60** and **61**), displayed antifouling activity against macro- and/or microfouling species [18]. The hybridization of intermediate chalcone with a glycosyl moiety was performed through the click chemistry reaction, obtaining a 1,2,3-triazole ring as a linker, which has been much highlighted as a promising strategy in medicinal chemistry, giving rise to hybrids with a wide range of biological activities [19]. Moreover, as the triazole ring was proven to be an effective scaffold for marine biofouling prevention, the use of flavonoids with a triazole ring could result in promising antifouling agents [20]. To assess their antifouling activity, the compounds were studied concerning their effect on the settlement of the macrofouling species *M. galloprovincialis* mussel larvae and on the growth of five biofilm-forming marine bacteria, namely *Vibrio harveyi*, *Cobetia marina*, *H. aquamarina*, *Pseudoalteromonas atlantica,* and *R. litoralis*. Among the tested compounds, chalcones **57** (EC_50_ = 3.28 µM; 2.43 µg/mL), **58** (EC_50_ = 18.10 µM; 5.44 µg/mL), and **59** (EC_50_ = 9.64 µM; 3.18 µg/mL) displayed effective anti-settlement activity (EC_50_ values < 25 µg/mL), with triazolyl glycosylated chalcone **57** being the most potent and having the highest therapeutic ratio (>60.98). Concerning the antimicrofouling activity, only propargylated chalcones **60** and **61** presented some activity towards the growth of *R. litoralis* (EC_30_ values of 135 and 83.5 µM, respectively). Later, chalcone **57** was shown to also be able to inhibit the biofilm formation of two strains of bacteria *S. aureus* [21]. The compounds showing the highest anti-settlement effect towards the mussel larvae (**57**–**59**) were further evaluated against the biofilm-forming marine diatom *Navicula* sp. Only chalcone **57** was able to display some activity against this microalga’s growth (EC_50_ = 41.76 µM; 30.94 µg/mL). An ecotoxicity assay performed against the marine non-target organism *Artemia salina* showed no toxicity for the chalcones **57**–**59** (less than 10% mortality at 50 μM) [18]. In this study, it was highlighted that the presence of a 3,4,5-trimethoxy group on the B ring of the chalcone scaffold seems to be beneficial for antifouling activity. Following the promising results obtained for compound **57**, the toxicity of compound **57** to humans was preliminarily assessed with an immortalized human retinal pigment epithelial cell line (hTERT-RPE-1) and compared with the commercial biocide Econea®. While the commercial biocide caused a 50% decrease in the cell viability at the lowest concentration tested (1 µM), chalcone **57** was only able to decrease the cell viability by 50% at the highest concentration tested (100 µM), which allows us to hypothesize that chalcone **57** may present a more safe profile [22]. Overall, the results obtained for triazole-linked chalcone glycoside **57** suggest that the conjugation of flavonoids with a glycosyl moiety through a click chemistry approach might be a potential tool for the development of new antifouling agents.

### 2.2. Flavonols

**Figure 4:**
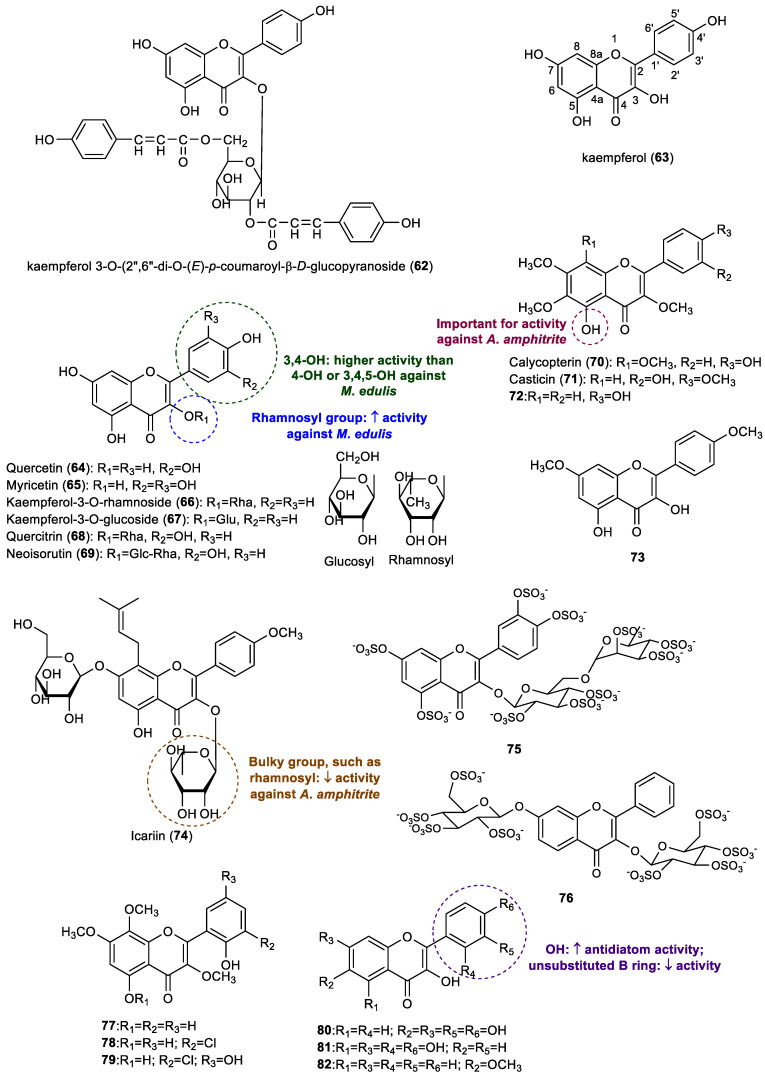
Structures of flavonols **62**–**82** with antifouling activity and some SAR considerations. Compounds **63**, **64**, and **77**–**79** were isolated from marine sources.

Flavonols comprise the second most reported class of flavonoids with an effect in the prevention of marine biofouling. The structures of flavonols with antifouling potential are presented in Figure 4.

The Kaempferol glucoside (kaempferol 3-*O*-(2″,6″-di-*O*-(*E*)-*p*-coumaroyl-β-d-glucopyranoside, **62**), isolated for the first time from the leaves of *Quercus dentata,* was the first flavonol reported for antifouling evaluation [13]. This flavonol showed high repellent activity against the settlement of blue mussel *Mytilus edulis* at 0.22 µmol/cm^2^, whereas the positive control, copper sulfate, showed similar activity at higher concentration (0.50 µmol/cm^2^), indicating a twofold more potent effect of this flavonol than copper sulfate. Authors also compared the activity of compound **62** with kaempferol (**63**) (concentration of 6.0 µmol/cm^2^ to exert the same activity) and *p*-coumaric acid (7.3 µmol/cm^2^ to exert the same activity), as well as a mixture of kaempferol and *p*-coumaric acid (1:2) (2.2 µmol/cm^2^ to exert the same activity), concluding that compound **62** presented higher activity than both kaempferol (**63**) and *p*-coumaric acid, thus suggesting that the combination of the kaempferol and *p*-coumaric acid moieties is associated with an improvement in antifouling activity [13].

Several flavonol aglycones, including kaempferol (**63**), quercetin (**64**), myricetin (**65**), and glycosides, such as kaempferol 3-*O*-rhamnoside (**66**), kaempferol 3-*O*-glucoside (**67**), quercitrin (**68**), neoisorutin (**69**), as well as kaempferol 3-*O*-(2″,6″-di-*O*-(*E*)-*p*-coumaroyl-β-d-glucopyranoside (**62**), previously reported by Yamashita et al. [13], were tested by Singh and collaborators for their antifouling potential against the settlement of blue mussel *M. edulis*, and their activity was compared with the standard antifouling compound copper sulfate [23]. Although these flavonols were obtained commercially for this study, these compounds are found in nature, namely in terrestrial and marine sources, and are associated with a wide range of biological activities [9,24,25,26,27]. Flavonoid **62** presented the highest activity in this study, with a more than twofold higher activity than the positive control copper sulfate. On the other hand, the other flavonoids presented only moderate activity, with 5% to 20% of the activity of copper sulfate. By comparing the activity of the tested flavonols, it appeared that flavonoid glycosides with a rhamnosyl moiety were more active than analogues with glycosyl moiety, and the presence of two hydroxy groups on the B ring allied to the two hydroxy groups on the A ring appeared to enhance the anti-settlement activity [23].

The antifouling activity of the hydroxylated flavonols kaempferol (**63**) and neoisorutin (**69**) was also reported by Zhou et al. [28]. Both compounds showed a promising effect against the larval settlement of the barnacle *Amphibalanus amphitrite*, displaying EC_50_ values of 33.54 μM (9.6 µg/mL) and 65.68 μM (40.1 µg/mL), respectively. These results reinforce the potential of these natural flavonols in the prevention of the settlement of macrofouling species. In addition to these flavonoids, calycopterin (**70**), casticin (**71**), 5,4′-dihydroxy-3,6,7-trimethoxyflavone (**72**), 3,5-dihydroxy-7,4′-dimethoxyflavone (**73**), and icariin (**74**) also behave as inhibitors of the larval settlement of the same barnacle [28] (EC_50_ values ranging from 7.26 μM to 57.17 μM, 2.5 µg/mL–35.3 µg/mL), with casticin (**71**, EC_50_: 8.01 μM, 3.0 µg/mL; LC_50_/EC_50_: >16.7) and 5,4′-dihydroxy-3,6,7-trimethoxyflavone (**72**, EC_50_: 7.26 μM, 2.5 µg/mL; LC_50_/EC_50_: >20.0) being the most active flavonols. All flavonols, **63**, **69**, and **70**–**74**, were obtained from terrestrial plants. The analysis of these results suggests that the presence of a hydroxy group at C5 is important for antifouling activity, whereas the presence of a bulky group such as rhamnosyl at C3 may reduce the activity [28].

In a study performed by Kong et al., the flavonols kaempferol (**63**) and quercetin (**64**), isolated from the methanol extract of the leaves of the marine halophyte *Apocynum venetum,* were tested for their antifouling activity against the marine fouling bacteria *Bacillus thuringiensis*, *Pseudoalteromonas elyakovii* and *Pseudomonas aeruginosa* [29]. At a concentration of 100 µg/disc, quercetin (**64**) (inhibition zone: 9.3–9.5 mm) and kaempferol (**63**) (inhibition zone: 9.5–17.4 mm) displayed moderate antibacterial activities against the three tested bacteria, compared to the positive control chloramphenicol at the same concentration (inhibition zone 29.2–33.7 mm) [29].

The effect of quercetin (**64**) on microfouling organisms was also studied by Gopikrishnan et al. [30]. This natural flavonol, isolated from the estuarine sediment actinobacterium *Streptomyces fradiae* PE7, was able to inhibit the growth of 12 bacterial strains isolated from marine fouling samples (*Staphylococcus* sp. M1, *Micrococcus* sp. M50, *Lactobacillus* sp. M6, *Bacillus* sp. N16, *Aeromonas* sp. N8, *Alcaligenes* sp. P2, *Alcaligenes* sp. N22, *Alcaligenes* sp. E4, *Vibrio* sp. M25, *Pseudomonas* sp. P1, *Pseudomonas* sp. N9, and *Kurthia* sp. P3), with MIC values ranging from 5.36 to 82.72 μM (1.62–25.0 µg/mL). This compound was also shown to inhibit the cyanobacterial *Anabaena* sp. and *Nostoc* sp. spore germination at 100 μg/mL and the adherence of the *Perna indica* mussel foot at 306 ± 19.6 μg/mL [30]. Interestingly, the compound quercetin (**64**) was tested in different studies against different marine organisms, namely a mussel species, marine bacteria, and algae, showing its potential to act against different levels of fouling community.

Our group also reported the synthesis and antifouling evaluation of a series of nature-inspired sulfated compounds, including flavonoids [31]. Among them, rutin persulfate (**75**) showed moderate anti-settlement activity towards plantigrades of the mussel *Mytilus galloprovincialis*, with an EC_50_ value of 22.59 μM and a LC_50_/EC_50_ ratio >22.13. Moreover, this flavonoid also displayed significant inhibitory activity against the growth of the marine bacteria *Vibrio harveyi* (EC_50_ value of 7.69 µM) and was shown to be non-toxic in *Artemia salina* and Luminescent *Vibrio fischeri* ecotoxicity assays. In this study, another sulfated flavonoid glycoside (compound **76**) was shown to moderately inhibit the growth of the marine bacteria *Halomonas aquamarina*, with an EC_50_ value of 42.3 µM [31].

Haider and colleagues tested the antifouling activity of a series of flavonoids against diatoms *Chaetoceros socialis*, *Navicula leavissima*, and *Navicula parva*, including seven flavonols, namely casticin (**71**) and flavonols **77**–**82** [32]. Flavonols **77**–**79** were isolated from the marine-derived fungus *Aspergillus candidus*, whereas the natural flavonoids, **80**, **81** (morin) and **82**, tested in this study were commercially obtained. All tested flavonols displayed antidiatom activity against *C. socialis* (EC_50_ 11.62–44.73 μM, 4.0–13.52 µg/mL). Regarding their activity against *N. leavissima*, compounds **71**, **80**, and **81** displayed activities with EC_50_ values ranging between 20.32 and 54.33 μM (6.14–16.42 µg/mL). Only flavonols **80**–**81** showed activity against *N. parva* (EC_50_ 18.76–20.38 μM, 5.67–6.16 µg/mL), whereas the commercial biocide Sea-Nine 211 showed activity against all strains of diatoms, with EC_50_ values ranging between 3.79 and 5.21 μM (1.07–1.47 µg/mL). Interestingly, these two flavonols with hydroxy groups as substituents on the B ring displayed activity against the three strains of diatoms tested, being the most promising compounds in the study, whereas an unsubstituted B ring leads to compound **82**, which has lower antidiatom activity [32]. 

### 2.3. Flavones

**Figure 5:**
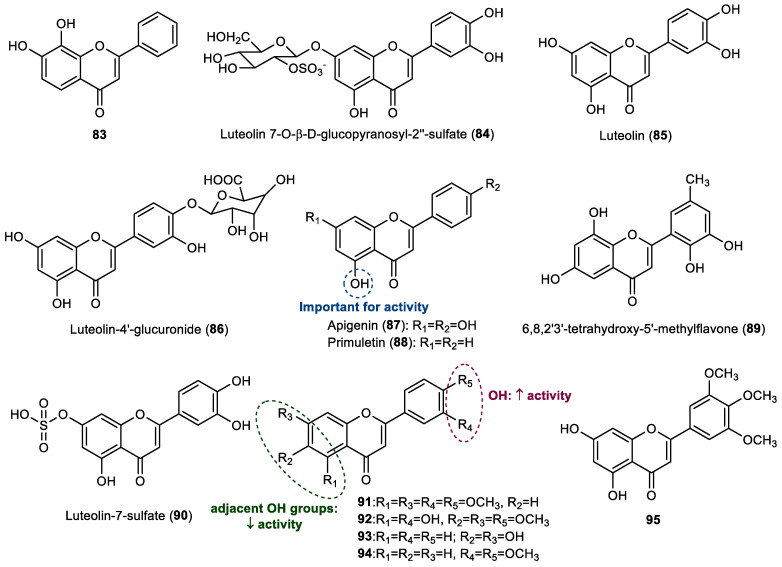
Structures of flavones **83**–**95** with antifouling activity and some SAR considerations. Compounds **84**–**86**, **89**, and **90** were isolated from marine sources.

Thirteen flavones were also found to exert some influence in the prevention of marine biofouling, being the structures of compounds presented in Figure 5.

Besides the evaluation of a series of flavonols, already reported in Section 2.2, Singh et al. also tested the activity of 7,8-dihydroxyflavone (**83**), known as tropoflavin, against the settlement of the blue mussel *M. edulis*. This compound showed 25% of the activity of the positive control copper sulfate [23]. Although this compound was commercially obtained for this study, tropoflavin occurs in nature, namely in terrestrial plants, and has been proven to exert therapeutic potential [33].

Jensen et al. observed that a lower number of thraustochytrid protists were observed to be associated with healthy leaf tissue in the marine angiosperm *Thalassia testudinum* than were found in sterilized samples, suggesting that *T. testudinum* may biosynthesize some metabolites which could be responsible for the low attachment of the thraustochytrid *Schizochytrium aggregatum* [34]. Therefore, the antifouling potential of *T. testudinum* extracts was evaluated and studies were performed aiming to identify the possible natural products responsible for the anti-settlement activity. It was demonstrated that extracts of *T. testudinum* leaf tissues inhibited the growth of the co-occurring thraustochytrid *S. aggregatum* and deterred the attachment of *S. aggregatum* motile zoospores to an extract-impregnated substrate. Bioassay-guided chemical fractionation of this extract allowed us to identify the flavone glycoside luteolin 7-*O*-*β*-d-glucopyranosyl-2″-sulfate (**84**), which was able to reduce the attachment of *S. aggregatum* zoospores, with an IC_50_ of 511.9 μM (270 µg/mL) [34]. These results support the hypothesis that luteolin 7-*O*-*β*-d-glucopyranosyl-2″-sulfate (**84**) could be responsible for the anti-settlement activity observed in the *Thalassia testudinum* extracts. 

Qi et al. reported the extraction and isolation of flavonoids from the South China Sea seagrass *Enhalus acoroides* [35]. Among them, the flavones luteolin (**85**) and luteolin-4′-glucuronide (**86**) showed some antibacterial activity against several strains of marine bacteria, with luteolin being active against *Loktanella hongkongensis*, *Pseudoalteromonas piscida*, *Rhodovulum* sp., and *Vibrio alginolyticus*, whereas luteolin-4′-glucuronide presented some activity against *L. hongkongensis*, *V. alginolyticus*, *Vibrio furnissii,* and *Vibrio halioticoli*. Moreover, luteolin 4′-glucuronide highly inhibited the larval settlement of *Bugula neritina* at a nontoxic concentration with an EC_50_ value of 1.12 μM (0.52 µg/mL) [35].

Later, luteolin (**85**), obtained from the terrestrial plant *Arachis hypogata,* as well as apigenin (**87**), obtained from terrestrial plant *Apium graveolens,* and the commercially obtained primuletin (**88**) were evaluated against the larval settlement of the barnacle *A. amphitrite* by Zhou et al., along with the flavonols reported in Section 2.2. [28]. Although primuletin was commercially obtained for this study, this flavone is found in nature, namely in terrestrial plants [36]. These compounds were shown to have a high activity, with EC_50_ values ranging from 11.47 to 13.28 μM (2.8–3.8 µg/mL). Considering that these compounds presented with similar activity, it seems that the presence of the hydroxy group at C5 is important for antifouling activity against barnacle larval settlement, whereas the presence of the other substituents is not essential for this activity [28].

The 6,8,2′3′-tetrahydroxy-5′-methylflavone (**89**), isolated from a broth of gorgonian coral-associated fungus *Penicillium* sp. SCSGAF 0023 by Bao et al., showed significant antifouling activity against the larval settlement of *A. amphitrite*, with an EC_50_ value of 22.35 μM (6.71 µg/mL) and low toxicity (LC_50_/EC_50_ ratio > 14.9) [37].

Considering the potential of the surface of the eelgrass Zostera marina to inhibit the colonization of microfouler organisms, Guan et al. performed an extraction of the *Z. marina* leaves in propanol and tested the extracted fractions against marine bacterial strains [38]. The authors identified the presence of the flavones luteolin (**85**) and luteolin-7-sulfate (**90**) in the extract which might be responsible for the activity. Furthermore, both flavones, luteolin (**85**) and luteolin-7-sulfate (**90**) were tested against the marine bacteria *Vibrio cyclitrophicus* and *Marivita litorea*. At concentration of 14.5 µg/mL, both luteolin (**85**) and luteolin-7-sulfate (**90**) exhibited comparable activity against both marine bacteria [38].

Besides the evaluation of flavonols **71** and **77**–**82** against diatoms *C. socialis*, *N. leavissima*, and *N. parva*, Haider et al. also studied the activity of the flavones luteolin (**85**) and flavones **91**–**94** against these biofouling organisms [32]. Luteolin (**85**) was the most promising flavone in this study, being active against all strains of diatoms tested (*N. leavissima:* EC_50_ = 30.39 μM, 8.70 µg/mL; *C. socialis*: EC_50_ = 21.59 μM, 6.18 µg/mL; and *N. parva*: EC_50_ = 27.15 μM, 7.77 µg/mL), whereas flavones **91**–**92** were active against *N. leavissima* (**91**: EC_50_ of 26.52 µM, 9.08 µg/mL; **92**: 58.32 µM, 20.08 µg/mL) and *C. socialis* (**91**: EC_50_ of 10.75 µM, 3.68 µg/mL; **92**: 25.15 µM, 8.66 µg/mL). Compound **93** was only active for *C. socialis* (EC_50_ 57.15 µM, 14.53 µg/mL), while compound **94** only showed activity against *N. leavissima* (EC_50_ of 55.19 µM, 15.58 µg/mL) [32]. The presence of hydroxy groups on the B ring seems to be favorable for antidiatom activity, as well as the substituents on the A ring, but the presence of adjacent hydroxy groups on the A ring is not beneficial.

Flavone tricetin 3′,4′,5′-trimethyl ether (**95**), synthesized by us, showed promising activity against the settlement of the mussel *M. galloprovincialis* larvae, with an EC_50_ value of 8.34 µM (2.87 µg/mL), and with low toxicity against the non-target marine organism *A. salina* [18]. In an attempt to study the mechanism of action of antifouling compounds, and based on the role of tyrosinase enzyme on the production of byssal threads by fouling organisms, an evaluation of the inhibition of this enzyme was performed, with flavone **95** able being to decrease tyrosinase activity, reaching 23.5% of inhibition at 100 µM [18].

### 2.4. Flavanones

**Figure 6:**
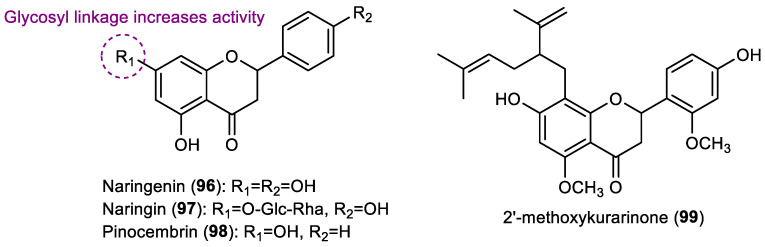
Structures of flavanones **96**–**99** with antifouling activity.

As far as we know, only four flavanones with antifouling activity have been reported so far (Figure 6).

Yoshioka et al. showed that the flavanone naringenin (**96**), isolated from the methanol extracts of the bark of *Prunus jamasakura* showed some repellent activity against the blue mussel *M. edulis* at a concentration of 1.2 mg/cm^2^, although the activity of this flavonoid is lower than the positive control, copper sulfate, at a concentration of 0.08 mg/cm^2^ [39].

Naringenin (**96**), as well as the flavanones naringin (**97**) (obtained commercially) and pinocembrin (**98**) (isolated from *Eucalyptus signata*), were evaluated against the settlement of blue mussel *M. edulis* by Singh et al. and the activity was compared with the standard antifouling copper sulfate [23]. In comparison with positive control, compounds **96**, **97** and **98** presented lower activity, having 11%, 24%, and 25% of the activity of the positive control copper sulfate, respectively. Naringin **97** showed to be more active than naringenin **96**, inferring the importance of glycosyl linkage for this activity [23].

The flavanone 2′-methoxykurarinone (**99**), isolated from the ethyl acetate extract of the Chinese herb *Sophora flavescens* by Feng and coworkers, was shown to strongly inhibit the settlement of cyprid larvae of *Balanus albicostatus* (EC_50_ of 4.46 µM, 2.02 µg/mL) by a non-toxic mechanism, since the LC_50_ value was higher than 25 µg/mL [40].

### 2.5. Isoflavones

**Figure 7:**
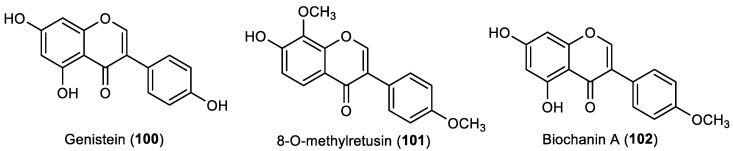
Structures of isoflavones **100**–**102** with antifouling activity. Isoflavone **101** was isolated from marine sources.

A total of three isoflavones with antifouling activity were described (Figure 7).

Besides the activity evaluation of the flavanone naringenin (**96**), Yoshioka et al. showed that the isoflavone genistein (**100**), also isolated from the methanolic extracts of the bark of *Prunus jamasakura*, exhibited some anti-settlement activity against the blue mussel *M. edulis*. Nevertheless, the activity of this isoflavone (9% of mussel settlement inhibition at 1.6 mg/cm^2^ concentration) was substantially lower when compared with the positive control copper sulfate (100% of mussel settlement inhibition at 0.08 mg/cm^2^ concentration) and also lower than the flavanone naringenin [39].

In addition to the evaluation of seven flavonols (Section 2.2), one flavone (Section 2.3) and three flavanones (Section 2.4), Singh and collaborators also tested the activity of isoflavone genistein (**100**) against the settlement of blue mussel *M. edulis*. This compound was found to display activity in the inhibition of the settlement of mussels, with 9% of the activity of the positive control copper sulfate, having a similar activity compared to the other flavonoids tested in the same study [23].

The antifouling activity of genistein (**100**), as well as the isoflavone 8-*O*-methylretusin (**101**), was also assessed by Zhou et al. The authors studied the effect of some flavonoids, including isoflavones genistein and 8-*O*-methylretusin, which were obtained by the purification of extracts of terrestrial plants *Genista tinctoria* and *Pueraria alopecuroides*, respectively, against the larval settlement of the barnacle *A. amphitrite* [28]. Genistein (**100**) presented strong activity (EC_50_: 11.10 µM, 3.0 µg/mL; LC_50_/EC_50_: >16.7), while isoflavone 8-*O*-methylretusin (**101**) presented with an EC_50_ value of 44.59 µM (13.3 µg/mL). Complementary assays in the field with the incorporation of the genistein in a resin-based paint showed that, after one month of the test panels being submerged, the density of barnacles was significantly lower when compared to the control panel tests. Moreover, some panels were coated with a mixture of genistein and a copper sulfate formulation, which showed lower densities of barnacles than the panels coated with the single compound after 3 months of submergence. Considering that the fate of the environment is a concern for the study of potential antifoulants, to evaluate the genistein behavior in seawater, hydrolysis assays were performed. Genistein seemed to be readily hydrolyzed in the marine environment, with the degradation speed being significantly affected by pH, namely in an alkaline environment, which should counteract the accumulation of high levels of this compound in marine environments [28].

In other study, it was reported that *O*-methylretusin (**101**), isolated from the methanolic extract of the leaves of the marine halophyte *Apocynum venetum*, presented activity against the marine fouling bacteria *B. thuringiensis* (inhibition zone 9.4 mm) and *P. aeruginosa* (inhibition zone 10.5 mm) at a concentration of 100 µg/disc, whereas the positive control chloramphenicol presented an inhibition zone of 33.7 and 30.3 mm at the same concentration [29]. These results suggest the potential of *O*-methylretusin (**101**) to prevent biofouling promoted by macro and microfouling agents. 

In addition to the evaluation of flavonols **71** and **77**–**82**, and flavones **81** and **91**–**94** described in Section 2.2 and Section 2.3, respectively, Haider and collaborators also tested the antifouling activity of the isoflavone biochanin A (**102**) against diatoms *C. socialis*, *N. leavissima*, and *N. parva* [32]. Although a commercial stock was used, this compound is found in nature, particularly in terrestrial plants, and display a wide range of biological activities [41]. This compound was able to inhibit the growth of diatom *C. socialis*, with an EC_50_ value of 28.60 µM (8.13 µg/mL), whereas the positive control Sea-Nine 211^®^ presented an EC_50_ value of 3.93 µM (1.11 µg/mL). Moreover, compared to the other flavonoids tested in the same study, biochanin A (**102**) was only active against one strain of diatom, as well as flavonoids **77**–**79**, **82**, and **93**–**94**, whereas the remaining flavonoids were active against two or three strains of diatoms [32].

### 2.6. Other Flavonoids

**Figure 8:**
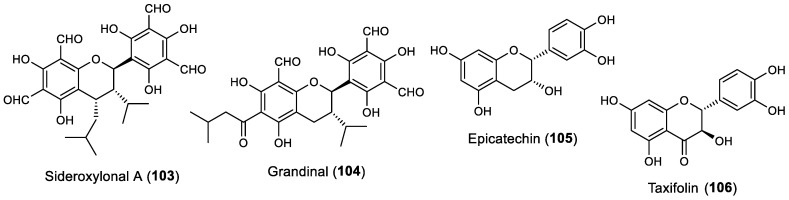
Structures of other classes of flavonoids with antifouling activity, including flavans **103**–**104**, flavanol **105**, and flavanonol **106**. Flavonoids **105** and **106** were isolated from marine sources.

Other flavonoids, including two flavans, one flavanol, and one flavanonol (Figure 8), also showed some potential in marine biofouling prevention and are reported below.

Sideroxylonal A (**103**), a flavan isolated from the leaves of *Eucalyptus grandis* by Singh et al., showed high repellent activity against blue mussel *M. edulis* at a concentration of 0.032 µmol/cm^2^ [42]. Another flavan, grandinal (**104**), isolated from the methanolic extract of the leaves of *E. grandis* by the same research group, also showed anti-settlement activity against the blue mussel *M. edulis*, comparable with the standard antifouling copper sulfate at the same concentrations tested [43]. Interestingly, the weaker activity of grandinal (**104**) compared with sideroxylonal A (**103**) may be due to the presence of tautomeric forms of grandinal. Grandinal was also found to exert some antibacterial activity against *Staphylococcus aureus* and *B. subtilis*, with an MIC of 50 and 100 µg/mL, respectively, which leads us to infer the dual activity of this molecule against both macrofouling and microfouling species [43]. 

In addition to the evaluation of flavonols **63** and **64**, and isoflavone **101** as referred to in Section 2.2 and Section 2.5, respectively, Kong et al. also studied the activity of the flavanol epicatechin (**105**), isolated from the methanolic extract of the leaves of the marine halophyte *Apocynum venetum,* against the marine fouling bacteria *B. thuringiensis*, *P. elyakovii,* and *P. aeruginosa*. At a concentration of 100 μg/disc, this compound showed some antibacterial activity against all the tested bacteria, with an inhibition zone diameter of 9.2–10.9 mm, whereas the positive control chloramphenicol presented an inhibition zone diameter of 29.2–33.7 mm at the same concentration. Moreover, compound **105** was shown to display a similar activity to flavonols **63** and **64**, whereas isoflavone **101** was only active against two strains of marine bacteria [29].

Gopikrishnan et al. reported the production and isolation of the flavanonol taxifolin (**106**) from the mangrove-derived actinobacterium *Streptomyces sampsonii* (PM33) [44]. After the isolation and characterization of this flavonoid, antifouling assays were performed. It was found that the taxifolin (**106**) is active against several biofouling bacterial strains, namely *Staphylococcus* sp. M1, *Micrococcus* sp. M50, *Lactobacillus* sp. M6, *Bacillus* sp. N16, *Aeromonas* sp. N8, *Alcaligenes* sp. P2, *Alcaligenes* sp. N22, *Alcaligenes* sp. E4, *Vibrio* sp. M25, *Pseudomonas* sp. P1, *Pseudomonas* sp. N9, and *Kurthia* sp. P3, with MIC values ranging from 5.32 to 82.17 µM; 1.62–25 μg/mL. Moreover, at a concentration of 100 μg/mL, this compound was shown to reduce the *Anabaena* sp. and *Nostoc* sp. spore germination by more than 70%. Taxifolin (**106**) was also shown to reduce the adherence of the mussel *Perna indica*. Furthermore, field experiments revealed the antifouling activity of taxifolin when tested on wooden surface and PVC panels. A toxicity assay using zebra fish model showed that in a concentration of 0.5 μg/mL and 1 μg/mL, taxifolin (**106**) caused pericardial edema in 0.6% of zebrafish embryos, while with an increase in the concentration to 1.5 μg/mL and 2.0 μg/mL, 60% of zebrafish embryos had pericardial edema. The cytotoxicity of taxifolin (**106**) was also assayed in vitro against the normal lung Bronchial Epithelial BEAS – 2B cell line, with LC_50_ values ranging between 150 μg/mL and 200 μg/mL [44].

## 3. Conclusions

The phenomenon of marine biofouling is very complex, involving a wide diversity of marine organisms and depending on multiple variables, and, generally, the screening studies performed englobe a few or even just one biofouling species, as described in this study.

In this review, a total of 106 flavonoids with antifouling activity against macro- and microfouling organisms were compiled by chemical classes. Although some flavonoids were only evaluated for their activity against one single species of fouling organisms, namely the mussel *M. galloprovincialis* or the barnacle *A. amphitrite*, some compounds were tested against several marine organisms, showing their potential to be effective in real scenarios of complex biofouling communities. For example, flavonol kaempferol (**63**) was found to display antibacterial activity against several marine bacteria and antifouling activity against two species of macrofouling organisms (mussel and barnacles) whereas quercetin (**64**) showed antibacterial activity and antifouling activity against a species of mussel. The flavone luteolin (**85**) was found to be active against marine bacteria, diatoms, and a macrofouling barnacle. Some flavonoids, namely the isoflavone genistein (**100**) and chalcones **4**, **45**, and **51**–**56**, were incorporated into marine coatings, showing interesting antifouling potential in field assays.

Several mechanisms of action have been associated with antifouling activity. However, only few studies reported the evaluation of the mechanism of action for antifouling flavonoids. Therefore, future assays should include the assessment of the mechanism involved in the antifouling activity, which could be beneficial in the design of more effective agents.

Considering the need of obtaining environmentally safe antifoulants, ecotoxicological assessments are needed. In fact, only a few flavonoids were assessed for their ecotoxicological behavior against marine non-target species, namely chalcones **48**–**50** and **57**–**59** and flavones **75** and **95**, which proved to be non-toxic against *A. salina*. In addition, studies of toxicity in human cell lines should be performed for the most promising antifouling compounds. In this review, only chalcone **57** was assayed against one human cell line and the potential for toxicity was compared with a commercial biocide. Chalcone **57** was shown to be non-toxic, whereas the commercial biocide displayed toxicity to the human cell line at low concentrations. 

The study of degradation of antifouling compounds is also important for understanding the long-term persistence of an antifouling compound in the environment. These assays should be assessed under representative conditions of pH, temperature, light, and microbial community [45]. It should be noted that only isoflavone genistein (**100**) was evaluated for its degradation behavior in a marine environment. 

Beyond the antifouling potential reported in this study, some flavonoids, including kaempferol (**63**), naringenin (**96**), genistein (**100**), quercetin (**64**), myricetin (**65**), luteolin (**85**), and apigenin (**87**), are known for their wide range of therapeutic activities including antimicrobial activity (antifungal, antibacterial) [46,47,48,49,50,51,52], which could suggest their potential to act against microorganisms, some of them also found in the marine environment.

Considering the feasible high-scale synthesis of flavonoids in the laboratory, allied with their high potential as antifoulants, this class of compounds could be considered for incorporation into antifouling paints.

## Data Availability

Not applicable.

## Supplementary Materials

The following supporting information can be downloaded at: https://www.mdpi.com/article/10.3390/md22020077/s1, Table S1: Structure, origin, and antifouling activity of flavonoids **1**–**106** reported in the review.
