# Supplementary material for: The Role of Natural and Synthetic Flavonoids in the Prevention of Marine Biofouling
Source: Mar Drugs. 2024 Feb 2;22(2):77. doi: 10.3390/md22020077 (PMC10889971; doi:10.3390/md22020077)
Supplement: Supplementary file 1 [file marinedrugs-22-00077-s001.zip › marinedrugs-2851485-supplementary.pdf]

Supplementary Material

The role of natural and synthetic flavonoids in the prevention of marine biofouling

Daniela Pereira [1,2], Madalena Pinto [1,2], Joana R. Almeida [2], Marta Correia-da-Silva [1,2,*] and Honorina Cidade [1,2,*]

[1] Laboratory of Organic and Pharmaceutical Chemistry, Department of Chemical Sciences, Faculty of Pharmacy of the University of Porto, Rua de Jorge Viterbo Ferreira 228, 4050-313 Porto, Portugal

[2] Interdisciplinary Centre of Marine and Environmental Research (CIIMAR), University of Porto, Edifício do Terminal de Cruzeiros do Porto de Leixões, Avenida General Norton de Matos, S/N, 4450-208 Matosinhos, Portugal

* Correspondence: m_correiadasilva@ff.up.pt (M.C.-d.-S.), hcidade@ff.up.pt (H.C.)

Table S1. Structure, origin, and antifouling activity of flavonoids **1-106** reported in the review.

Chalcones			
**Code and structure of Flavonoid**	**Origin**	**Activity**	**Reference**
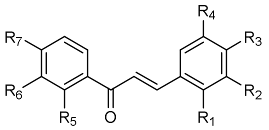<br><br>**1**: $R_1=R_2=R_4=R_5=R_6=R_7=H$; $R_3=NMe_2$<br>**2**: $R_1=R_2=R_4=R_5=R_6=R_7=H$; $R_3=SMe$<br>**3**: $R_1=R_2=R_4=R_5=R_6=H$; $R_3=SMe$; $R_7=NO_2$<br>**4**: $R_1=R_2=R_3=R_4=R_5=R_6=R_7=H$<br>**5**: $R_1=R_2=R_4=R_5=R_7=H$; $R_3=SMe$; $R_6=OH$<br>**6**: $R_1=R_2=R_4=R_5=R_6=H$; $R_3=SMe$; $R_7=OMe$<br>**7**: $R_1=R_2=R_4=R_6=R_7=H$; $R_3=SMe$; $R_5=OH$<br>**8**: $R_1=R_2=R_4=R_5=R_6=H$; $R_3=SMe$; $R_7=Me$<br>**9**: $R_1=R_2=R_4=R_5=R_6=H$; $R_3=SMe$; $R_7=Cl$<br>**10**: $R_1=R_2=R_4=R_5=R_7=H$; $R_3=SMe$; $R_6=NO_2$<br>**11**: $R_1=R_2=R_4=R_5=R_6=R_7=H$; $R_3=OMe$<br>**12**: $R_1=R_2=R_4=R_6=H$; $R_3=SMe$; $R_5=R_7=Cl$<br>**13**: $R_1=OMe$; $R_2=R_3=R_4=R_5=R_6=R_7=H$<br>**14**: $R_1=R_2=R_4=R_5=R_6=H$; $R_3=SMe$; $R_7=Br$<br>**15**: $R_1=R_5=R_6=R_7=H$; $R_3=R_2=R_4=OMe$<br>**16**: $R_1=R_2=R_4=R_5=R_6=H$; $R_3=SMe$; $R_7=OH$<br>**17**: $R_1=R_2=R_6=H$; $R_3=R_4=OMe$; $R_5=R_7=Cl$<br>**18**: $R_2=R_4=R_5=R_7=H$; $R_1=R_3=OMe$; $R_6=NO_2$<br>**19**: $R_1=R_2=R_5=R_7=H$; $R_3=R_4=OMe$; $R_6=NO_2$<br>**20**: $R_1=R_2=R_4=R_5=R_7=H$; $R_3=NMe_2$; $R_6=NO_2$<br>**21**: $R_1=OMe$; $R_2=R_3=R_4=R_5=R_6=H$; $R_7=NO_2$<br>**22**: $R_1=R_2=R_4=R_6=H$; $R_3=NMe_2$; $R_5=R_7=Cl$<br>**23**: $R_1=R_2=R_4=R_5=R_7=H$; $R_3=OMe$; $R_6=OH$<br>**24**: $R_1=OMe$; $R_2=R_3=R_4=R_5=R_7=H$; $R_6=NO_2$<br>**25**: $R_1=R_3=R_4=R_5=H$; $R_2=NO_2$; $R_6\text{-}R_7=\text{-O-CH}_2\text{-O-}$<br>**26**: $R_1=R_2=R_4=R_5=R_7=H$; $R_3=Cl$; $R_6=NO_2$<br>**27**: $R_1=R_3=R_4=R_5=R_7=H$; $R_2=NO_2$; $R_6=Br$<br>**28**: $R_1=Cl$; $R_2=R_3=R_4=R_5=R_7=H$; $R_6=OMe$<br>**29**: $R_1=Cl$; $R_2=R_3=R_4=R_5=R_6=H$; $R_7=OEt$<br>**30**: $R_1=R_2=R_4=R_5=R_6=R_7=H$; $R_3=SO_2CH_3$<br>**31**: $R_1=R_2=R_4=R_5=R_6=H$; $R_3=SO_2CH_3$; $R_7=NO_2$<br>**32**: $R_1=R_2=R_4=R_5=R_6=H$; $R_3=SO_2CH_3$; $R_7=Cl$<br>**33**: $R_1=R_2=R_4=R_6=H$; $R_3=SO_2CH_3$; $R_5=R_7=Cl$<br>**34**: $R_1=R_5=R_7=Cl$; $R_2=R_3=R_4=R_6=H$<br>**35**: $R_1=Cl$; $R_2=R_3=R_4=R_5=R_7=H$; $R_6=NO_2$<br>**36**: $R_1=Cl$; $R_2=R_3=R_4=R_5=R_7=H$; $R_6=OH$<br>**37**: $R_1=R_2=R_4=R_5=H$; $R_3=SMe$; $R_6\text{-}R_7=\text{-O-CH}_2\text{-O-}$<br>**38**: $R_1=R_2=R_4=R_5=R_7=H$; $R_3=SMe$; $R_6=NH_2$<br>**39**: $R_1=R_2=R_4=R_5=H$; $R_3=SMe$; $R_6=R_7=OMe$<br>**40**: $R_1=R_2=R_4=R_5=R_7=H$; $R_3=SMe$; $R_6=OMe$<br>**41**: $R_1=R_2=R_4=R_5=R_6=H$; $R_3=SMe$; $R_7=OEt$<br>**42**: $R_1=R_2=R_4=R_5=R_7=H$; $R_3=SMe$; $R_6=Br$<br>**43**: $R_1=R_2=R_4=R_5=R_6=H$; $R_3=SMe$; $R_7=F$<br>**44**: $R_1=Cl$; $R_2=R_3=R_4=R_5=H$; $R_6\text{-}R_7=\text{-O-CH}_2\text{-O-}$<br>**45**: $R_1=OMe$; $R_2=R_3=R_4=R_6=H$; SMe; $R_5=R_7=Cl$<br>**46**: $R_1=R_2=R_4=R_5=H$; $R_3=OMe$; $R_6\text{-}R_7=\text{-O-CH}_2\text{-O-}$<br>**47**: $R_1=R_2=R_4=R_5=H$; $R_3=OH$; $R_6\text{-}R_7=\text{-O-CH}_2\text{-O-}$	Synthesis	- *Bacillus flexus*: MIC = 0.002 – 0.466 μM<br>- *Pseudomonas fluorescens*: MIC = 0.004 – 0.133 μM<br>- *Vibrio natriegens*: MIC = 0.024 – 0.249 μM	(Sivakumar, Prabhawathi et al. 2010)
	Synthesis	Reduction of the adhesion of the marine bacterium *V. natriegens* for marine paints with chalcone **45** comparing to control paint and paint with copper: Polymer surfaces coated with the paint without additives exhibited an amount of colony forming unit (CFU) of 30 – 40×10[6]/mL after 28 days of assay, followed by surfaces with paint mixed with copper (20 – 40×10[6]/mL) and the surfaces with chalcone **45** mixed paint (1×10[6]/mL).	(Sivakumar, Prabhawathi et al. 2010)
	Synthesis	Paints containing chalcone **4** (1.25 mmol chalcone/100 g of paint) were able to inhibit the settlement and growth of diatoms *Achnanthes* sp., *Coscinodiscus* sp., *Grammatophora* sp., *Licmophora* sp., *Navicula* sp., *Nitzschia longissima*, *Pinnularia* sp., *Pleurosigma* sp., *Synedra* sp. and *Skeletonema costatum*, and protozoans such as *Vorticella* sp. and *Zoothamnium* sp. by about 70% in comparison with control paint.	(Sathicq, Paola et al. 2019)

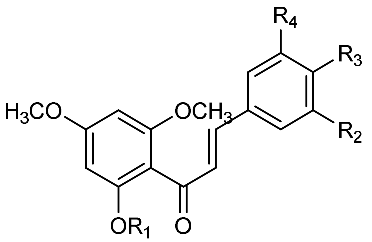 **48**: R_1=R_2=R_4=H, R_3=OCH_3 **49**: R_1=R_3=R_4=H, R_2=OCH_3 **50**: R_1=Prenyl, R_2=R_3=R_4=OCH_3	Synthesis	**48**: *Mytilus galloprovincialis* mussel larvae, EC_{50}: 34.63 μM **49**: *M. galloprovincialis* (EC_{50}: 7.24 μM); bacterial growth inhibitory activity against *Halomonas aquamarina* (EC_{50}: 18.67 μM) and *Roseobacter litoralis* (EC_{50}: 4.09 μM) **50**: *M. galloprovincialis* (EC_{50}: 16.48 μM); growth inhibitory activity against *H. aquamarina* (EC_{50}: 18.78 μM) and *R. litoralis* (EC_{50}: 12.34 μM); diatom inhibitory activity against *Cylindrotheca* sp. (EC_{50}: 7.04 μM), *Halamphora* sp. (EC_{50}: 14.65 μM), *Nitzschia* sp. (EC_{50}: 20.31 μM) and *Navicula* sp. (EC_{50}: 6.75 μM)	(Almeida, Moreira et al. 2018)
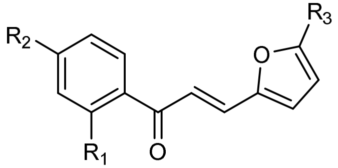 **51**:R_1=R_2=R_3=H **52**:R_1=R_2=H; R_3=Me **53**:R_1=R_3=H; R_2=Me **54**:R_1=H; R_2=R_3=Me **55**:R_1=OH; R_2=R_3=H **56**:R_1=OH; R_2=H; R_3=Me	Synthesis	Paints containing chalcones **51-56** (1.25 mmol chalcone/100 g of paint) were able to inhibit the settlement and growth of diatoms *Achnanthes* sp., *Coscinodiscus* sp., *Grammatophora* sp., *Licmophora* sp., *Navicula* sp., *Nitzschia longissima*, *Pinnularia* sp., *Pleurosigma* sp., *Synedra* sp. and *Skeletonema costatum*, and protozoans *Vorticella* sp. and *Zoothamnium* sp. by about 60-80% and the settlement of filamentous red alga *Polysiphonia* sp., green alga *Ulva* sp., and filamentous brown alga *Ectocarpus* sp. and calcareous tubeworms *Hydroides* sp. and *Spirorbis* sp. by about 75-~100% in comparison with control paint.	(Sathicq, Paola et al. 2019)

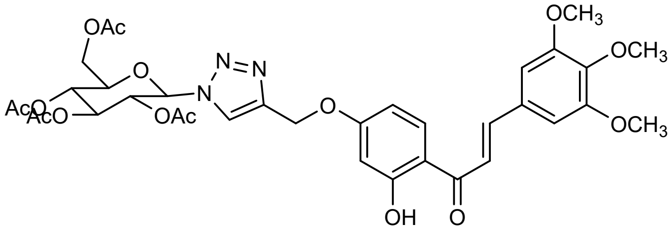<br>**57**	Synthesis	Activity against mussel *M. galloprovincialis* (EC_{50} = 3.28 μM, 2.43 μg/mL).<br>Activity against diatom *Navicula* sp. (EC_{50} = 41.76 μM, 30.94 μg/mL)	(Pereira, Gonçalves et al. 2021)
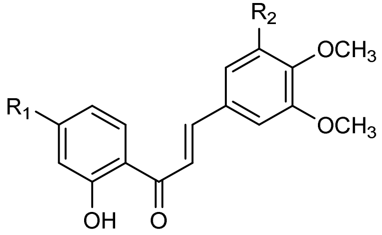<br>**58**: R_1=OH, R_2=H<br>**59**: R_1=OH, R_2=OCH_3<br>**60**: R_1=OCH_2CCH, R_2=H<br>**61**: R_1=OCH_2CCH, R_2=OCH_3	Synthesis	**58**: activity against *M. galloprovincialis* (EC_{50} = 18.10 μM, 5.44 μg/mL).<br>**59**: activity against *M. galloprovincialis* (EC_{50} = 9.64 μM, 3.18 μg/mL).<br>**60**: bacterial inhibitory activity against *R. litoralis* (EC_{50} = 135 μM).<br>**61**: bacterial inhibitory activity against *R. litoralis* (EC_{50} = 83.5 μM).	(Pereira, Gonçalves et al. 2021)
**Flavonols**			
**Code and structure of Flavonoid**	**Origin**	**Activity**	**Reference**
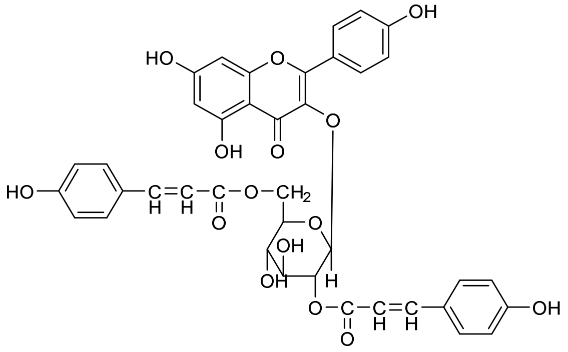<br>kaempferol 3-O-(2'',6''-di-O-(*E*)-*p*-coumaroyl-β-*D*-glucopyranoside (**62**)	Isolated from the leaves of *Quercus dentata*	Anti-settlement activity against the blue mussel *Mytilus edulis* at 0.22 μmol/cm², higher than positive control copper sulfate (0.50 μmol/cm²)	(Yamashita, Etoh et al. 1989)

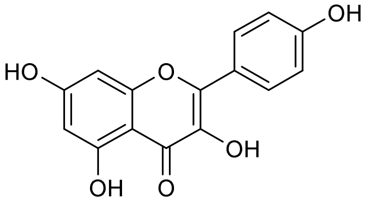<br>kaempferol (**63**)	Commercial	Anti-settlement activity against the blue mussel *M. edulis* at 6.0 μmol/cm², lower than positive control copper sulfate (0.50 μmol/cm²)	(Yamashita, Etoh et al. 1989)
	Commercial	Low anti-settlement activity against blue mussel *M. edulis* (8% of positive control copper sulfate).	(Singh, Etoh et al. 1997)
	Isolated from the terrestrial plant *Micromelum integerrinum*	Anti-settlement activity against the barnacle *Amphibalanus amphitrite* larvae (EC_{50}: 33.54 μM, 9.6 μg/mL; $LC_{50}/EC_{50} > 5.2$)	(Zhou, Zhang et al. 2009)
	Isolated from the methanol extract of the leaves of the marine halophyte *Apocynum venetum*	Activity against marine bacteria *Bacillus thuringiensis*, *Pseudoalteromonas elyakovii* and *Pseudomonas aeruginosa* at a concentration of 100 μg/disc (inhibition zone 9.5 – 17.4 mm), (positive control chloramphenicol at 100 μg/disc: inhibition zone 29.2 – 33.7 mm)	(Kong, Fang et al. 2014)
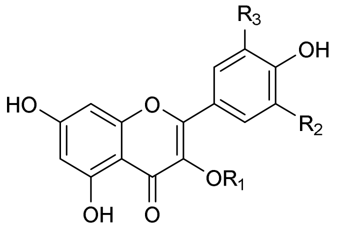<br>Quercetin (**64**): R_1=R_3=H, R_2=OH	Commercial	Low anti-settlement activity against blue mussel *M. edulis* (12% comparing to positive control copper sulfate).	(Singh, Etoh et al. 1997)
	Isolated from the methanol extract of the leaves of the halophyte *Apocynum venetum*	Activity against *B. thuringiensis*, *P. elyakovii* and *P. aeruginosa* at 100 μg/disc (inhibition zone 9.3 – 9.5 mm) (positive control chloramphenicol at 100 μg/disc: inhibition zone 29.2 – 33.7 mm)	(Kong, Fang et al. 2014)
	Isolated from the marine-derived actinobacterium, *Streptomyces fradiae* PE7	Activity against marine bacteria *Staphylococcus* sp. M1 (MIC: 5.36 μM, 1.62 μg/mL), *Micrococcus* sp. M50 (MIC: 10.34 μM, 3.125 μg/mL), *Lactobacillus* sp. M6 (MIC: 10.34 μM, 3.125 μg/mL), *Bacillus* sp. N16 (MIC: 5.36 μM, 1.62 μg/mL), *Aeromonas* sp. N8 (MIC: 20.68 μM, 6.25 μg/mL), *Alcaligenes* sp. P2 (MIC: 20.68 μM, 6.25 μg/mL), *Alcaligenes* sp. N22 (MIC: 20.68 μM, 6.25 μg/mL), *Alcaligenes* sp. E4	(Gopikrishnan, Radhakrishnan et al. 2016)

		(MIC: 20.68 μM, 6.25 μg/mL), *Vibrio* sp. M25 (MIC: 41.36 μM, 12.5 μg/mL), *Pseudomonas* sp. P1 (MIC: 82.72 μM, 25 μg/mL), *Pseudomonas* sp. N9 (MIC: 82.72 μM, 25 μg/mL) and *Kurthia* sp. P3 (MIC: 41.36 μM, 12.5 μg/mL);<br>Inhibition of the *Anabaena* sp. and *Nostoc* sp. spore germination at 100 μg/mL and the adherence of the Perna indica mussel foot at 306 ± 19.6 μg/mL	
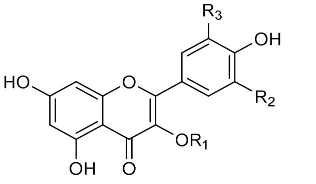<br>Myricetin (**65**): R_1=H, R_2=R_3=OH	Commercial	Low anti-settlement activity against *M. edulis* (8% comparing to positive control copper sulfate).	(Singh, Etoh et al. 1997)
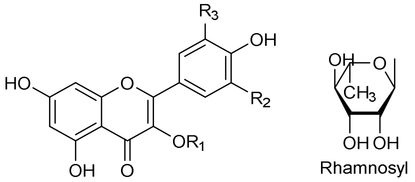<br>Kaempferol-3-*O*-rhamnoside (**66**): R_1=Rha, R_2=R_3=H	Commercial	Anti-settlement activity against *M. edulis* (10% of positive control copper sulfate).	(Singh, Etoh et al. 1997)
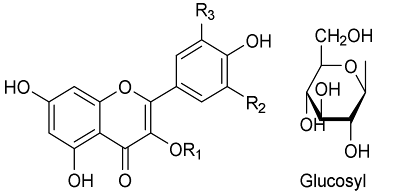<br>Kaempferol-3-O-glucoside (**67**): R_1=Glu, R_2=R_3=H	Commercial	Low anti-settlement activity against mussel *M. edulis* (5% of positive control copper sulfate).	(Singh, Etoh et al. 1997)

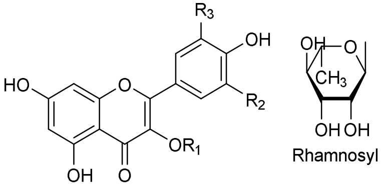 Quercitrin (**68**): R_1=Rha, R_2=OH, R_3=H	Commercial	Anti-settlement activity against mussel *M. edulis* (20% of positive control copper sulfate).	(Singh, Etoh et al. 1997)
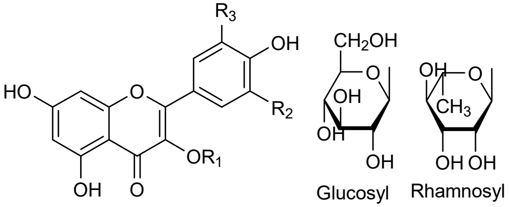 Neoisorutin (**69**): R_1=Glc-Rha, R_2=OH, R_3=H	Commercial	Anti-settlement activity against mussel *M. edulis* (19% of positive control copper sulfate).	(Singh, Etoh et al. 1997)
	Isolated from the terrestrial plant *Micromelum integerrinum*	Barnacle *A. amphitrite* larvae (EC_{50}: 65.68 μM, 40.1 μg/mL; LC_{50}/EC_{50} > 5.0)	(Zhou, Zhang et al. 2009)
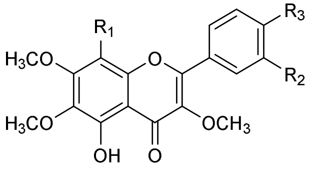 Calycopterin (**70**): R_1=OCH_3, R_2=H, R_3=OH	Isolated from the terrestrial plant *Micromelum integerrinum*	Barnacle *A. amphitrite* larvae (EC_{50}: 9.08 μM, 3.4 μg/mL; LC_{50}/EC_{50} > 14.7)	(Zhou, Zhang et al. 2009)
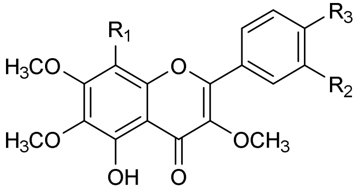 Casticin (**71**): R_1=H, R_2=OH, R_3=OCH_3	Isolated from the terrestrial plant *Micromelum integerrinum*	Barnacle *A. amphitrite* larvae (EC_{50}: 8.01 μM, 3.0 μg/mL; LC_{50}/EC_{50} > 16.7)	(Zhou, Zhang et al. 2009)
	Commercial	Antidiatom activity against *N. leavissima* (EC_{50}: 38.65 μM, 14.47 μg/mL) and *C. socialis* (EC_{50}: 19.53 μM, 7.31 μg/mL)	(Haider, Ma et al. 2020)
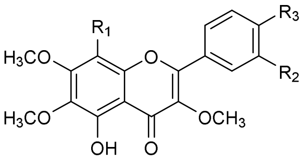 **72**:R_1=R_2=H, R_3=OH	Isolated from the terrestrial plant *Micromelum integerrinum*	Barnacle *A. amphitrite* (EC_{50}: 7.26 μM, 2.5 μg/mL; LC_{50}/EC_{50} > 20.0)	(Zhou, Zhang et al. 2009)

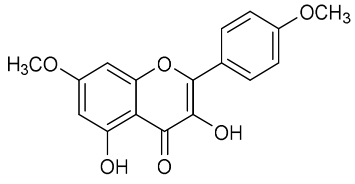 **73**	Isolated from the plant *Clausena dunniana*	Barnacle *A. amphitrite* (EC_{50}: 37.54 μM, 11.8 μg/mL; $LC_{50}/EC_{50} > 4.2$)	(Zhou, Zhang et al. 2009)
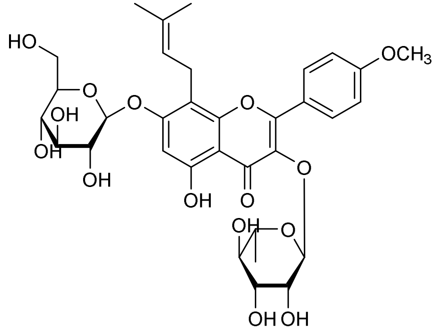 Icariin (**74**)	Isolated from the terrestrial plant *Epimedium segittatum*	Barnacle *A. amphitrite* (EC_{50}: 52.17 μM, 35.3 μg/mL; $LC_{50}/EC_{50} > 2.8$)	(Zhou, Zhang et al. 2009)
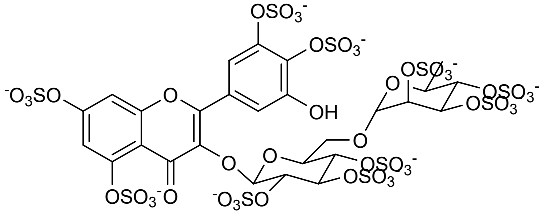 Rutin persulfate (**75**)	Synthesis	Mussel *M. galloprovincialis*, EC_{50}: 22.59 μM; LC_{50}/EC_{50}: >22.13. Inhibitory activity against marine bacteria *Vibrio harveyi* (EC_{50}: 7.69 μM)	(Almeida, Correia-da-Silva et al. 2017)
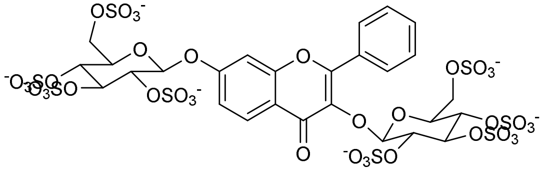 **76**	Synthesis	Inhibitory activity against marine bacteria *Halomonas aquamarina* (EC_{50}: 42.3 μM)	(Almeida, Correia-da-Silva et al. 2017)

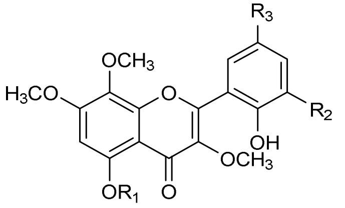<br>**77**:$R_1=R_2=R_3=H$<br>**78**:$R_1=R_3=H$; $R_2=Cl$<br>**79**:$R_1=H$; $R_2=Cl$; $R_3=OH$	Isolated from the marine-derived fungus *Aspergillus candidus*	Antidiatom activity against *C. socialis*:<br>**77**: EC_{50}: 11.62 μM, 4.0 μg/mL)<br>**78**: EC_{50}: 16.63 μM, 6.3 μg/mL)<br>**79**: EC_{50}: 18.75 μM, 7.4 μg/mL)	(Haider, Ma et al. 2020)
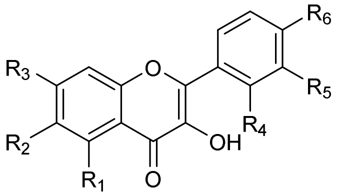<br>**80**:$R_1=R_4=H$; $R_2=R_3=R_5=R_6=OH$<br>**81**:$R_1=R_3=R_4=R_6=OH$; $R_2=R_5=H$<br>**82**:$R_1=R_3=R_4=R_5=R_6=H$; $R_2=OCH_3$	Commercial	**80**: activity against diatoms *N. leavissima* (EC_{50}: 54.33 μM, 16.42 μg/mL), *C. socialis* (EC_{50}: 44.73 μM, 13.52 μg/mL) and *N. parva* (EC_{50}: 18.76 μM, 5.67 μg/mL)<br>**81**: activity against diatoms *N. leavissima* (EC_{50}: 20.32 μM, 6.14 μg/mL), *C. socialis* (EC_{50}: 19.52 μM, 5.90 μg/mL) and *N. parva* (EC_{50}: 20.38 μM, 6.16 μg/mL)<br>**82**: activity against diatom *C. socialis* (EC_{50}: 37.54 μM, 10.07 μg/mL)	(Haider, Ma et al. 2020)
**Flavones**			
**Code and structure of Flavonoid**	**Origin**	**Activity**	**Reference**
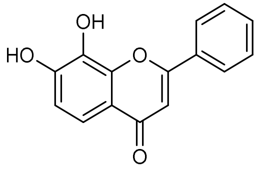<br>**83**	Commercial	Mussel *M. edulis* (25% of the activity of positive control copper sulfate)	(Singh, Etoh et al. 1997)
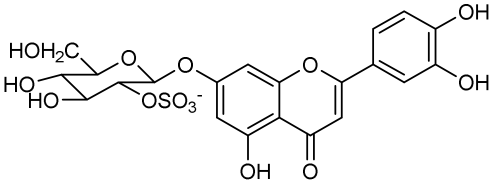<br>Luteolin 7-O-β-D-glucopyranosyl-2''-sulfate (**84**)	Isolated from the leaf tissue of the marine angiosperm *Thalassia testudinum*	Reduction of the attachment of thraustochytrid *Schizochytrium aggregatum* zoospores (IC_{50}: 511.9 μM, 270 μg/mL)	(Jensen, Jenkins et al. 1998)

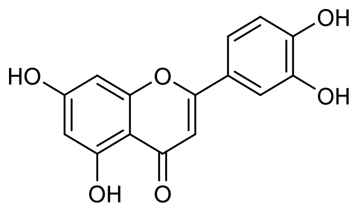<br>Luteolin (**85**)	Isolated from the South China Sea seagrass *Enhalus acoroides*	Activity against marine bacteria *Loktanella hongkongensis* (MIC: 174.68 μM, 50 μg/mL); low inhibition for bacteria *Pseudoalteromonas piscida*, *Rhodovulum* sp. and *Vibrio alginolyticus*	(Qi, Zhang et al. 2008)
	Isolated from the terrestrial plant *Arachis hypogata*	Activity against barnacle *A. amphitrite* larvae (EC_{50}: 13.28 μM, 3.8 μg/mL; LC_{50}/EC_{50} > 13.2)	(Zhou, Zhang et al. 2009)
	Isolated from an extract of the leaves of eelgrass *Zostera marina*	Inhibition of the settlement of marine bacteria *Vibrio cyclitrophicus* and *Marivita litorea* at 14.5 μg/mL	(Guan, Parrot et al. 2017)
	Commercial	Activity against diatoms *N. leavissima* (EC_{50}: 30.39 μM, 8.70 μg/mL), *C. socialis* (EC_{50}: 21.59 μM, 6.18 μg/mL) and *N. parva* (EC_{50}: 27.15 μM, 7.77 μg/mL)	(Haider, Ma et al. 2020)
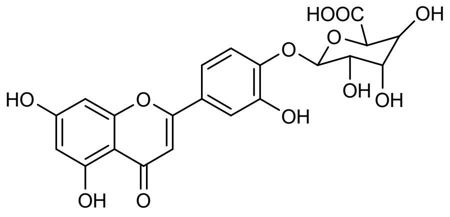<br>Luteolin-4'-glucuronide (**86**)	Isolated from the South China Sea seagrass *Enhalus acoroides*	Activity against marine bacteria *L. hongkongensis*, *V. alginolyticus*, *Vibrio furnissii* and *Vibrio halioticoli*. Inhibition of *Bugula neritina* larval settlement (EC_{50}: 1.12 μM, 0.52 μg/mL)	(Qi, Zhang et al. 2008)
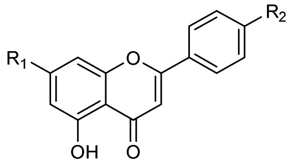<br>Apigenin (**87**): R_1=R_2=OH	Isolated from the terrestrial plant *Apium graveolens*	Activity against barnacle *A. amphitrite* (EC_{50}: 11.47 μM, 3.1 μg/mL; LC_{50}/EC_{50}> 16.1)	(Zhou, Zhang et al. 2009)
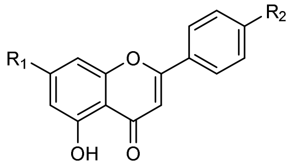<br>Primuletin (**88**): R_1=R_2=H	Commercial	Activity against barnacle *A. amphitrite* (EC_{50}: 11.75 μM, 2.8 μg/mL; LC_{50}/EC_{50}> 17.9)	(Zhou, Zhang et al. 2009)

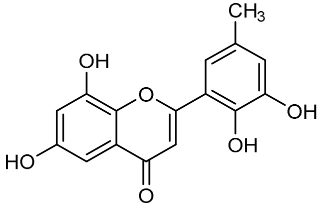 6,8,2'3'-tetrahydroxy-5'-methylflavone (**89**)	Isolated from a broth of gorgonian coral-associated fungus *Penicillium* sp. SCSGAF 0023	Activity against barnacle *A. Amphitrite* (EC_{50} value of 22.35 μM, 6.71 μg/mL; LC_{50}/EC_{50} ratio >14.9)	(Bao, Sun et al. 2013)
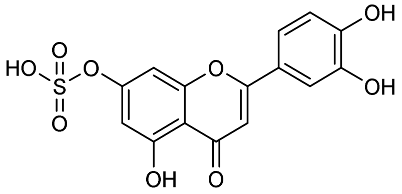 Luteolin-7-sulfate (**90**)	Isolated from an extract of the leaves of eelgrass *Zostera marina*	Inhibition of the settlement of marine bacteria *V. cyclitrophicus* and *M. litorea* at 14.5 μg/mL	(Guan, Parrot et al. 2017)
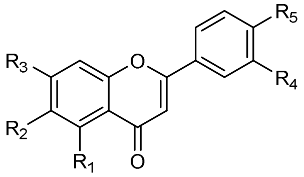 **91**:$R_1=R_3=R_4=R_5=OCH_3$, $R_2=H$<br>**92**:$R_1=R_4=OH$, $R_2=R_3=R_5=OCH_3$<br>**93**:$R_1=R_4=R_5=H$; $R_2=R_3=OH$<br>**94**:$R_1=R_2=R_3=H$, $R_4=R_5=OCH_3$	Commercial	**91**: active against diatoms *N. leavissima* (EC_{50}: 26.52 μM, 9.08 μg/mL) and *C. socialis* (EC_{50}: 10.75 μM, 3.68 μg/mL)<br>**92**: active against diatoms *N. leavissima* (EC_{50}: 58.32 μM, 20.08 μg/mL) and *C. socialis* (EC_{50}: 25.15 μM, 8.66 μg/mL)<br>**93**: active against diatom *C. socialis* (EC_{50}: 57.15 μM, 14.53 μg/mL)<br>**94**: active against diatom *N. leavissima* (EC_{50}: 55.19 μM, 15.58 μg/mL)	(Haider, Ma et al. 2020)
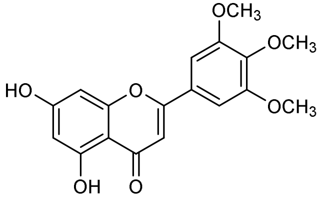 **95**	Synthesis	Activity against mussel *M. galloprovincialis* mussel larvae (EC_{50}: 8.34 μM, 2.87 μg/mL)	(Pereira, Gonçalves et al. 2021)
**Flavanones**			

Code and structure of Flavonoid	Origin	Activity	Reference
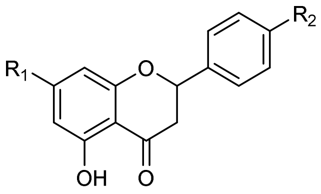 Naringenin (**96**): R_1=R_2=OH	Isolated from the methanol extract of the bark of *Prunus jamasakura*	Low anti-settlement activity against *M. edulis* at 1.2 mg/cm² concentration (11% of the activity of positive control copper sulfate at 0.08 mg/cm² concentration)	(Yoshioka, Etoh et al. 1990)
	Commercial	11% activity against mussel *M. edulis* comparing to positive control copper sulfate	(Singh, Etoh et al. 1997)
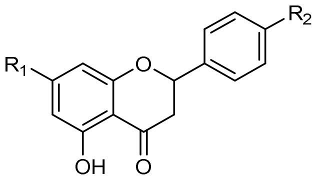 Naringin (**97**): R_1=O-Glc-Rha, R_2=OH	Commercial	24% activity against mussel *M. edulis* comparing to positive control copper sulfate	(Singh, Etoh et al. 1997)
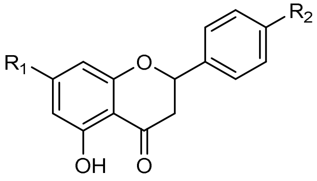 Pinocembrin (**98**): R_1=OH, R_2=H	Isolated from *Eucalyptus signata*	25% activity against mussel *M. edulis* comparing to positive control copper sulfate	(Singh, Etoh et al. 1997)
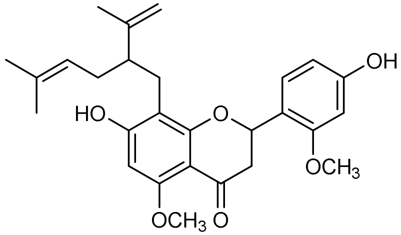 2'-methoxykurarinone (**99**)	Isolated from the ethyl acetate extract of the Chinese herb *Sophora flavescens*	Anti-settlement activity against *Balanus albicostatus* (EC_{50}: 4.46 μM, 2.02 μg/mL; LC_{50} value > 25 μg/mL)	(Feng, Ke et al. 2009)
**Isoflavones**			
**Code and structure of Flavonoid**	**Origin**	**Activity**	**Reference**

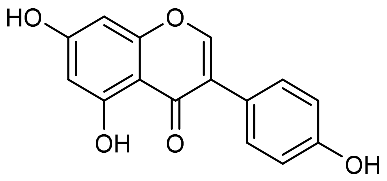 Genistein (**100**)	Isolated from the bark of *Prunus jamasakura*	Low anti-settlement activity against *M. edulis* at 1.6 mg/cm^2 concentration (9% of the activity of positive control copper sulfate at a concentration of 0.08 mg/cm^2)	(Yoshioka, Etoh et al. 1990)
	Commercial	Low anti-settlement activity against *M. edulis* (9% of the activity comparing to positive control copper sulfate)	(Singh, Etoh et al. 1997)
	Isolated from the plant *Genista tinctoria*	Anti-settlement activity against barnacle *A. amphitrite* (EC_{50}: 11.10 μM, 3.0 μg/mL; LC_{50}/EC_{50}: >16.7)<br>Density of barnacles in panels coated with paint containing genistein was significantly lower after one month of a field assay when compared with the control panel tests.	(Zhou, Zhang et al. 2009)
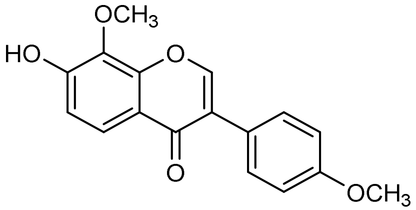 8-O-methylretusin (**101**)	Isolated from the plant *Pueraria alopecuroides*	Anti-settlement activity against barnacle *A. amphitrite* (EC_{50}: 44.59 μM, 13.3 μg/mL)	(Zhou, Zhang et al. 2009)
	Isolated from the methanol extract of the leaves of the marine halophyte *Apocynum venetum*	Activity against bacteria *B. thuringiensis* (inhibition zone 9.4 mm) and *P. aeruginosa* (inhibition zone 10.5 mm) at a concentration of 100 μg/disc, the positive control chloramphenicol: inhibition zone of 33.7 and 30.3 mm at the same concentration	(Kong, Fang et al. 2014)
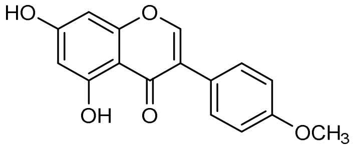 Biochanin A (**102**)	Commercial	Activity against diatom *C. socialis* (EC_{50}: 28.60 μM, 8.13 μg/mL)	(Haider, Ma et al. 2020)
**Other flavonoids**			
**Code and structure of Flavonoid**	**Origin**	**Activity**	**Reference**

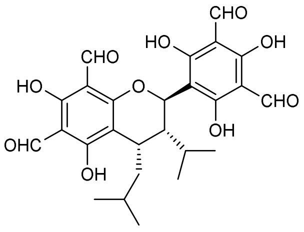<br>Sideroxylonal A (**103**)	Isolated from the leaves of *Eucalyptus grandis*	High activity against mussel *M. edulis* at a concentration of 0.032 μmol/cm^2	(Singh, Takahashi et al. 1996)
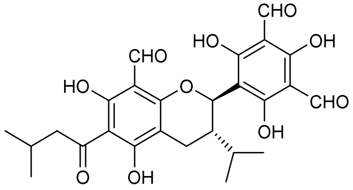<br>Grandinal (**104**)	Isolated from the methanolic extract of the leaves of *E. grandis*	Activity against mussel *M. edulis* (same activity as the positive control copper sulfate)	(Singh, Hayakawa et al. 1997)
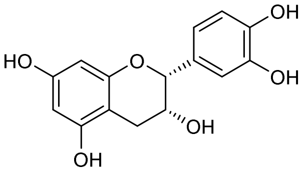<br>Epicatechin (**105**)	Isolated from the methanol extract of the leaves of the marine halophyte *Apocynum venetum*	Activity against bacteria *B. thuringiensis* (inhibition zone: 9.2 mm), *P. elyakovii* (inhibition zone: 10.6 mm) and *P. aeruginosa* (inhibition zone: 10.9 mm) at 100 μg/disc (positive control chloramphenicol: inhibition zone 29.2 – 33.7 mm at the same concentration)	(Kong, Fang et al. 2014)
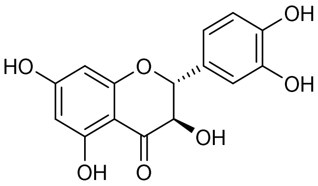<br>Taxifolin (**106**)	Isolated from the mangrove derived actinobacterium *Streptomyces sampsonii* (PM33)	- Activity against bacteria *Staphylococcus* sp. M1 (MIC: 5.32 μM, 1.62 μg/mL), *Micrococcus* sp. M50 (MIC: 10.27 μM, 3.125 μg/mL), *Lactobacillus* sp. M6 (MIC: 10.27 μM, 3.125 μg/mL), *Bacillus* sp. N16 (MIC: 5.32 μM, 1.62 μg/mL), *Aeromonas* sp. N8 (MIC: 20.54 μM, 6.25 μg/mL), *Alcaligenes* sp. P2 (MIC: 20.54 μM, 6.25 μg/mL), *Alcaligenes* sp. N22 (MIC: 20.54 μM, 6.25 μg/mL), *Alcaligenes* sp. E4 (MIC: 20.54 μM, 6.25 μg/mL), *Vibrio* sp. M25 (MIC: 41.08 μM, 12.5 μg/mL), *Pseudomonas* sp. P1 (MIC: 82.17 μM, 25 μg/mL), *Pseudomonas* sp. N9 (MIC:	(Gopikrishnan, Radhakrishnan et al. 2019)

		82.17 μM, 25 μg/mL) and *Kurthia* sp. P3 (MIC: 41.08 μM, 12.5 μg/mL);<br>- Reduction of more than 70% of the *Anabaena* sp. and *Nostoc* sp. spore germination at 100 μg/mL;<br>- Decrease of the foot adherence of mussel *Perna indica*.	

Almeida, J. R., M. Correia-da-Silva, E. Sousa, J. Antunes, M. Pinto, V. Vasconcelos and I. Cunha (2017). "Antifouling potential of Nature-inspired sulfated compounds." Scientific Reports **7**(1): 42424.

Almeida, J. R., J. Moreira, D. Pereira, S. Pereira, J. Antunes, A. Palmeira, V. Vasconcelos, M. Pinto, M. Correia-da-Silva and H. Cidade (2018). "Potential of synthetic chalcone derivatives to prevent marine biofouling." Science of The Total Environment **643**: 98-106.

Bao, J., Y.-L. Sun, X.-Y. Zhang, Z. Han, H.-C. Gao, F. He, P.-Y. Qian and S.-H. Qi (2013). "Antifouling and antibacterial polyketides from marine gorgonian coral-associated fungus Penicillium sp. SCSGAF 0023." The Journal of Antibiotics **66**(4): 219-223.

Feng, D. Q., C. H. Ke, C. Y. Lu and S. J. Li (2009). "Herbal plants as a promising source of natural antifoulants: evidence from barnacle settlement inhibition." Biofouling **25**(3): 181-190.

Gopikrishnan, V., M. Radhakrishnan, T. Shanmugasundaram, R. Pazhanimurugan and R. Balagurunathan (2016). "Antibiofouling potential of quercetin compound from marine-derived actinobacterium, Streptomyces fradiae PE7 and its characterization." Environmental Science and Pollution Research **23**(14): 13832-13842.

Gopikrishnan, V., M. Radhakrishnan, T. Shanmugasundaram, M. P. Ramakodi and R. Balagurunathan (2019). "Isolation, characterization and identification of antibiofouling metabolite from mangrove derived Streptomyces sampsonii PM33." Scientific Reports **9**(1): 12975.

Guan, C., D. Parrot, J. Wiese, F. D. Sönnichsen, M. Saha, D. Tasdemir and F. Weinberger (2017). "Identification of rosmarinic acid and sulfated flavonoids as inhibitors of microfouling on the surface of eelgrass Zostera marina." Biofouling **33**(10): 867-880.

Haider, W., J. Ma, X.-M. Hou, M.-Y. Wei, J.-Y. Zheng and C.-L. Shao (2020). "Natural Flavones and their Preliminary Structure–Antifouling Activity Relationship." Chemistry of Natural Compounds **56**(2): 334-337.

Jensen, P. R., K. M. Jenkins, D. Porter and W. Fenical (1998). "Evidence that a New Antibiotic Flavone Glycoside Chemically Defends the Sea Grass Thalassia testudinumagainst Zoosporic Fungi." Applied and Environmental Microbiology **64**(4): 1490-1496.

Kong, N.-N., S.-T. Fang, Y. Liu, J.-H. Wang, C.-Y. Yang and C.-H. Xia (2014). "Flavonoids from the halophyte Apocynum venetum and their antifouling activities against marine biofilm-derived bacteria." Natural Product Research **28**(12): 928-931.

Pereira, D., C. Gonçalves, B. T. Martins, A. Palmeira, V. Vasconcelos, M. Pinto, J. R. Almeida, M. Correia-da-Silva and H. Cidade (2021) "Flavonoid Glycosides with a Triazole Moiety for Marine Antifouling Applications: Synthesis and Biological Activity Evaluation." Marine Drugs **19** DOI: 10.3390/md19010005.

Qi, S.-H., S. Zhang, P.-Y. Qian and B.-G. Wang (2008). "Antifeedant, antibacterial, and antilarval compounds from the South China Sea seagrass Enhalus acoroides." Botanica Marina **51**(5): 441-447.

Sathicq, Á., A. Paola, M. Pérez, O. Dallesandro, M. García, J. P. Roldán, G. Romanelli and G. Blustein (2019). "Furylchalcones as new potential marine antifoulants." International Biodeterioration & Biodegradation **143**: 104730.

Singh, I. P., H. Etoh, E. Asai, K. Kikuchi, K. Ina, K. Koyasu and Y. Terada (1997). "Flavonoids and Stilbenes as Repellents against the Blue Mussel, Mytilus edulis galloprovincialis." Natural Product Sciences **3**(1): 49-54.

Singh, I. P., R. Hayakawa, H. Etoh, M. Takasaki and T. Konoshima (1997). "Grandinal, a New Phloroglucinol Dimer from Eucalyptus grandis." Bioscience, Biotechnology, and Biochemistry **61**(5): 921-923.

Singh, I. P., K. Takahashi and H. Etoh (1996). "Potent Attachment-inhibiting and -promoting Substances for the Blue Mussel, Mytilus edulis galloprovincialis, from Two Species of Eucalyptus." Bioscience, Biotechnology, and Biochemistry **60**(9): 1522-1523.

Sivakumar, P. M., V. Prabhawathi and M. Doble (2010). "2-Methoxy-2′,4′-dichloro chalcone as an antimicrofoulant against marine bacterial biofilm." Colloids and Surfaces B: Biointerfaces **81**(2): 439-446.

Sivakumar, P. M., V. Prabhawathi and M. Doble (2010). "Antibacterial activity and QSAR of chalcones against biofilm-producing bacteria isolated from marine waters." SAR and QSAR in Environmental Research **21**(3-4): 247-263.

Yamashita, N., H. Etoh, K. Sakata, A. Yagi, H. Ina and K. Ina (1989). "An Acylated Kaempferol Glucoside Isolated from Quercus dentata as a Repellent against the Blue Mussel Mytilus edulis." Agricultural and Biological Chemistry **53**(5): 1383-1385.

Yoshioka, A., H. Etoh, A. Yagi, K. Sakata and K. Ina (1990). "Isolation of Flavonoids and Cerebrosides from the Bark of Prunus jamasakura as Repellents against the Blue Mussel, Mytilus edulis." Agricultural and Biological Chemistry **54**(12): 3355-3356.

Zhou, X., Z. Zhang, Y. Xu, C. Jin, H. He, X. Hao and P.-Y. Qian (2009). "Flavone and isoflavone derivatives of terrestrial plants as larval settlement inhibitors of the barnacle Balanus amphitrite." Biofouling **25**(1): 69-76.
